# Improving GHG inventories by regional information exchange: a report from Asia

**DOI:** 10.1186/1750-0680-1-9

**Published:** 2006-08-23

**Authors:** Chisa Umemiya

**Affiliations:** 1 National Institute for Environmental Studies, 16-2 Onogawa, Tsukuba, Ibaraki, 305-8506, Japan

## Abstract

**Background:**

The Parties to the United Nations Framework Convention on Climate Change (UNFCCC) are required to develop and report a national inventory of greenhouse gases not controlled by the Montreal Protocol. In the Asia region, "Workshops on Greenhouse Gas Inventories in Asia (WGIA)" have been organised annually since 2003 under the support of the government of Japan. WGIAs promote information exchange in the region to support countries' efforts to improve the quality of greenhouse gas inventories. This paper reports the major outcomes of the WGIAs and discusses the key aspects of information exchange in the region for the improvement of inventories.

**Results:**

The major outcomes of WGIAs intended to help countries improve GHG inventories, can be summarised as follows: (1) identification of common issues and possible solutions by sector, (2) reporting country inventory practices, and (3) verification of the UNFCCC reporting requirements.

**Conclusion:**

The workshops provided the opportunity for countries to share common issues and constraints pertinent to GHG inventories and to exchange information regarding possible solutions for those issues based on their own experience. The relevance of information exchange is determined due to emission sources, emitting mechanisms from sources, and technologies used. Information exchange about emission sources that are unique to Asia, like those of the agriculture sector, contributes significantly to the accumulation of knowledge at the regional and global levels. Enabling countries to verify their national circumstances with the reporting requirements under UNFCCC is also an essential part of the WGIA information exchange activities.

## Background

The United Nations Framework Convention on Climate Change (UNFCCC) requires that all parties to the Convention report a national inventory of greenhouse gas emissions and removals following the guidelines of the Intergovernmental Panel on Climate Change (IPCC) with different reporting requirements for developed (Annex I) and developing (non-Annex I) countries [1-4]. The IPCC guidelines provide large volumes of estimation methods including detailed "activity data" which are essentially production and consumption activities and per unit greenhouse gas multipliers called "emission factors" [5-7]. The guidelines also provide "default emission factors" and "default activity data" to enable all countries to construct inventories, regardless of the lack of actual country-specific information. The guidelines encourage parties to make efforts to develop their own emission factors and activity data wherever it is possible since the default factors and activity data are known to deviate from the actual condition of emissions in a given country [2,3,8]. Developing country-specific emission factors and activity data has been a tough challenge particularly for non-Annex I countries where resources are more limited [8].

Given that most non-Annex I nations reported their first inventories in initial national communications and are now on their way to submitting subsequent national communications, it is highly likely that countries will be keen on improving the quality of greenhouse gas inventories and developing their own emission factors and activity data. Therefore, the demand for assistance for non-Annex I countries to improve their inventories is likely to rise and support for such improvements should be effectively made.

Owing to varied national conditions, countries differ in the intensity of their inventory efforts. Ecological, cultural, and social similarities in a region mean that the inventories of countries in the same region possess some common features. Therefore, information exchange among countries in the same region could be an effective way to support efforts to improve the quality of their greenhouse gas inventories.

Since 2003 "Workshops on Greenhouse Gas Inventories in Asia (WGIA)" have been organised annually under the support of the government of Japan [9-11]. The Asian countries participating are: Cambodia, China, India, Indonesia, Japan, the Republic of Korea, Lao PDR, Malaysia, Mongolia, Philippines, Thailand, and Vietnam. As a general rule, each country provides a pair of participants: one government official and one researcher involved in the inventory development. The meetings also benefited from the participation of representatives of international organisations, such as the UNFCCC Secretariat.

In this paper, in addition to reporting the major outcomes of WGIA, I will discuss the key aspects of information exchange activities in Asia to enhance the capacities of countries to improve the quality of inventories.

## Discussion

The major outcomes of WGIAs can be classified into three: (1) Identification of common issues and possible solutions by sector, (2) exchange of countries' practices, and (3) verification with the UNFCCC reporting requirements.

### Identification of common issues and possible solutions, by sector

In the 3^rd ^WGIA, participants were divided into four sectoral groups of energy; agriculture; Land Use, Land-Use Change and Forestry (LULUCF); and waste. The common issues of inventory development and possible solutions for those issues are summarised in Table 1[11].

**Table 1:** List of common issues and possible solutions, by sector [11]

Sector	Types	Identified common issues	Suggested possible solutions
Energy	Activity data	Collection of data	- Sharing experiences specifically relevant to the areas of transportaion, power plant, and heavy industry
	Emission factors	Development of improved emission factors	- Making tables for the values of local emission factors in Asia with assumptions adopted
	Quality assuarance and quality control (QA/QC)	Implementation of QA/QC	- Creating different database for comparison - Recording routine process and assignments of each implementing agency
	Uncertainty assessment	Implementation of uncertainty assessment	- Following up and updating data and information
Agriculture	Emission factors	Development of regional specific emission factors	- Implementing regional research projects - Enhancing collaboration among experts - Sharing database and experience
	Monitoring	Establishment of network for GHG emissions monitoring	
	Financial resource	Acquisition of funding for research and capacity development activities	
Land Use, Land-Use Change and Forestry (LULUCF)	Activity data	Adjustment of different level of details of forest categories and strata between states and provinces	- Encouraging local research agencies and universities to conduct research and receive endorsement from relevant authority for the work
	Emission (Removal) factors	Determination of the appropriate number of destructive sampling that is cost-effective	- Obtaining additional data from other information sources, such as national inventories of other countries in the region
	Uncertainty	Development of good activity data and emission factors for the key categories, which may be cost-ineffective	- Applying the *IPCC Good Practice Guidance and Uncertainty Management in National Greenhouse Gas Inventories *in regions and/or provinces that significantly contribute to the emissions of the categories
	Institutional arrangements	Frequent change of personnel engaged in inventory development	- Institutionalising inventory work, at least at the level of national focal point - Developing manuals in local language
Waste	Activity data	Collection of data, such as accurate waste generation amounts and degraded organic carbon	- Encouraging local research agencies and universities to do site measurements in waste areas and to collect detailed information of waste treatment management levels - Seeking support from local/relevant authorities for inventory work (etc.)
	Emission factors	Development of improved emission factors in different countries or regions	- Getting additional data from other information sources, such as national inventories of other countries in the region (etc.)
	Uncertainty	Development of reliable activity data and emission factors, which can reduce uncertainty	- Applying the *IPCC Good Practice Guidance and Uncertainty Management in National Greenhouse Gas Inventories *in regions and/or provinces that significantly contribute to the emissions of the categories (etc.)
	Institutional arrangements	Inconsistency of working groups in the waste sector at the domestic and international levels	- Developing manuals in local languages - Establishing an international network for cooperating in the waste sector (etc.)

Frequently identified issues across the sectors were those related to emission factors, activity data, and the uncertainty of estimated emissions. To address the common issues, the energy and agriculture sectoral groups suggested using the value of the regional network to share experiences among countries and create the regional database or table for emission factors. In the LULUCF and waste sectors, participating experts proposed a wide range of possible solutions that countries could implement on their own. I consider this variation in the discussion results among the sectors the simple reflection of these particular discussions and not dependent on the nature of inventory development in each sector.

### Exchange of country practices

Countries exchanged detailed information of country practices for developing and improving inventories. The participants generally agreed that sharing information of their practices was useful to better prepare their inventories, except in those few cases where specific emitting sources are irrelevant for certain countries (e.g. Mongolia has no emissions from rice cultivation because it does not cultivate rice domestically) or technologies or experimental systems introduced are too advanced thus could not be applied in some countries.

Moreover, it is likely that the degree of the usefulness of information exchange regarding country practices can be varied among different sectors, depending on the commonalities seen in the development of inventories for each sector. Experts on the agriculture sector reported that they considered sharing information about country practices across the region to be highly valuable, because some agricultural practices, such as rice cultivation and agroforestry adapted for the local conditions, are unique to the region and are not often seen outside the region [11]. The inventory development of the waste sector, on the other hand, is more controlled by the particular waste management system employed in each country, with huge variation. Hence, the waste group pointed out that the exchange of information on country practices, which cover the common aspects of inventory development (e.g. the implementation of measurement of methane emissions from landfills) should be selectively conducted [11].

### Verification with the UNFCCC reporting requirements

Countries also exchanged general information about national circumstances. By doing so, countries could verify whether the methodologies they use are adequate in the context of the UNFCCC requirements. Verifying and making sure that countries fully go along with the rules produces two results: not only fulfilling the reporting requirements, but also avoiding unnecessary efforts caused by misunderstanding or lack of awareness of some of the rules. Some participating countries were not aware of the rules that they are required to follow, a situation that was corrected by the representative from the UNFCCC Secretariat [11].

## Conclusion

Based on the experience of WGIA, the key aspects of information exchange activities in Asia to improve the quality of inventories may be summarised as follows:

i) Countries in the region share common issues and constraints to improve inventories, therefore, they can effectively exchange information about possible solutions for those issues based on their own experience.

ii) Information exchange can be effectively made if it focuses on the areas relevant to the majority of participating countries. The relevance can be according to emission sources, emitting mechanism from sources, and technologies used (e.g. experimental equipment for measurement of emissions).

iii) Information exchange about emission sources unique to Asia, e.g. those of the agricultural sector, should be encouraged as it will contribute to the accumulation of scientific and technical knowledge of Asia and the world.

iv) Verification of national circumstances with the UNFCCC reporting requirements is fundamental as such an opportunity can help countries satisfy those requirements.

## Competing interests

The author(s) declares that she has no competing interests.
